# Sertraline Hydrochloride Overdose Resulting in Diabetes Insipidus: A Case Report

**DOI:** 10.7759/cureus.60952

**Published:** 2024-05-23

**Authors:** Aifeng He, Leiming Xu, Suhui Li, Jinxin Qiu

**Affiliations:** 1 Emergency Medicine, Binhai County People’s Hospital Affiliated to Kangda College of Nanjing Medical University, Yancheng, CHN

**Keywords:** bidirectional affective disorder, pituitrin, antidiuretic hormone, diabetes insipidus, sertraline hydrochloride poisoning

## Abstract

Sertraline hydrochloride belongs to the selective serotonin reuptake inhibitor class of antidepressants, which can cause respiratory depression, hypotension, malignant vomiting, liver function impairment, and other symptoms when taken in excess. To our knowledge, reports of sertraline hydrochloride overdose causing diabetes insipidus in patients are rare. This report describes a unique case of a 17-year-old female patient who developed diabetes insipidus after a one-time oral intake of 20 sertraline hydrochloride tablets (50 mg/tablet) during the later course of treatment. Her symptoms were effectively relieved after treatment with pituitrin.

## Introduction

Sertraline hydrochloride, a class of antidepressants, is a selective serotonin reuptake inhibitor (SSRI) whose mechanism of action is related to the inhibition of serotonin reuptake in central neurons, enhancing the effect of serotonin in vivo. However, its effect on norepinephrine and dopamine reuptake is weak [1]. Sertraline hydrochloride is a hepatic metabolism drug combined with albumin and A-acid glycoprotein in vivo, with a binding rate of approximately 98%, the peak plasma concentration is reached six to eight hours after administration, a half-life of approximately 22-36 hours, a bioavailability of 88%, and relatively high safety [2]. Excessive intake of serotonin may cause different side effects, such as QT prolongation in the electrocardiogram, tip-twisting ventricular tachycardia, drowsiness, gastrointestinal discomfort (e.g., nausea and vomiting), tachycardia, tremor, and dizziness, with diabetes insipidus rarely reported [3,4].

Diabetes insipidus is a disease characterized by hypoosmotic polyuria, often secondary to abnormal synthesis, regulation, or renal action of antidiuretic hormones [5]. Inappropriate use of ketamine can induce the secretion of vasopressin, which leads to hyponatremia and hypoosmotic urine, and it is proposed that ketamine centrally stimulates the release of antidiuretic hormone from the hypothalamus [6]. Clinical studies have also found rare adverse complications of increased antidiuretic hormone secretion during the use of the antibiotic ciprofloxacin [7], with the main clinical symptoms being polyuria (i.e., a 24-hour urine volume of >40 mL/kg), polydipsia, and hypoosmotic urine [8]. At present, the main treatment methods for diabetes insipidus caused by abnormal antidiuretic hormone are hormone replacement therapy with medications such as pituitrin and desmopressin acetate.

Improper vasopressin secretion caused by sertraline hydrochloride is a syndrome that is rarely described in the literature. Knowledge of this adverse effect caused by excessive intake of sertraline hydrochloride comes mainly from case reports. At present, there is no specific treatment for excessive sertraline hydrochloride. The medical record we are sharing primarily discusses the rare occurrence and treatment process of diabetes insipidus in a 17-year-old girl with bipolar affective disorder, resulting from mood swings and an oral overdose of sertraline hydrochloride.

## Case presentation

A 17-year-old female patient became unconscious after orally ingesting 20 sertraline hydrochloride tablets (50 mg/tablet), and was found by her family and immediately brought to the emergency department of Binhai County People’s Hospital. She had a history of bipolar affective disorder and was taking the following oral medications: sertraline hydrochloride tablets, sodium valproate extended-release tablets, and quetiapine fumarate tablets. One hour before admission, she was found by her family to be unconscious, accompanied by convulsions of the limbs, beside a large amount of vomit (stomach contents) and an empty box of sertraline hydrochloride tablets, and was immediately admitted to our hospital.

On admission, the patient had a heart rate of 136 beats/minute, a respiration rate of 14 breaths/minute, a blood pressure of 84/56 mmHg, a finger-pulse oximetry of 91%, and a Glasgow Coma Scale (GCS) score of 5. Her pupils were equal in size and roundness bilaterally, with a diameter of 3.5 mm, and her light reflex was sluggish. The respiratory examination showed mild cyanosis of the lips and mouth, along with coarse breath sounds in both lungs. No other obvious positive signs were observed. The emergency physician immediately administered oxygen, rehydration and volume expansion, vasoactive drugs to raise blood pressure, gastric lavage, and other related treatments. Considering the patient’s critical condition, it was recommended to continue her treatment in the emergency intensive care unit, to which she was admitted.

**Figure 1:**
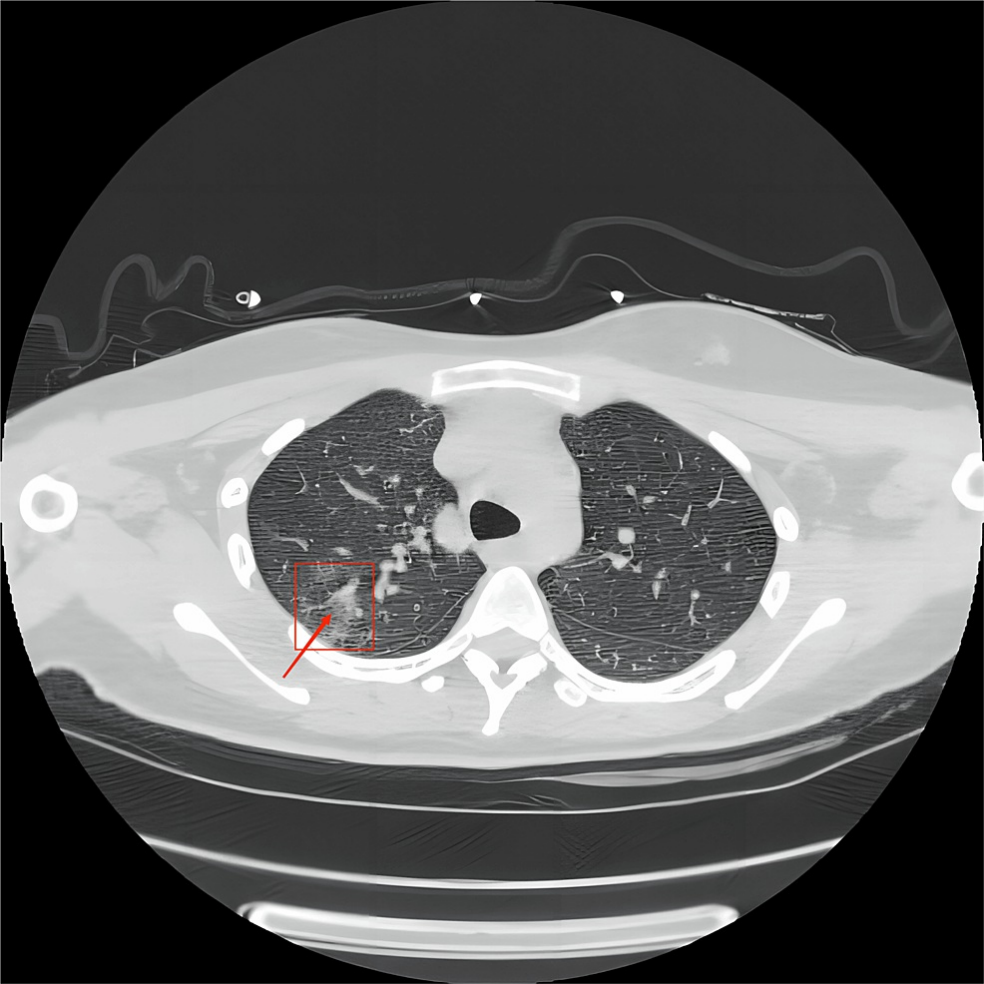
Chest computed tomography scan indicating exudative changes in the right lung with possible inflammation.

The patient exhibited a progressive decline in pulse oximetry, down to a minimum of 82%. Blood gas analysis showed the following (Table 1): pH of 6.92, partial pressure of oxygen at 85 mmHg, partial pressure of carbon dioxide at 63 mmHg, lactate at 14.6 mmol/L, and bicarbonate at 10 mmol/L. Chest computed tomography suggested exudative changes in the right upper lung with inflammation, as shown in Figure 1. The patient was immediately put on mechanical ventilation to maintain respiration, and sodium bicarbonate was given to correct acidosis. Norepinephrine was given to maintain blood pressure after deep vein placement. After accessing the femoral vein, the patient received hemoperfusion and additional bedside blood purification treatments to remove toxins and purify the blood. In addition, the patient received antibiotics to prevent infection, with fasting, gastrointestinal decompression, and catheter therapy. As a result of treatment, her symptoms were significantly reduced, her acid-base disorder was almost completely corrected, and her circulation was significantly improved.

**Table 1:** Laboratory investigation of the patient. PaO_2_ = partial pressure of oxygen; PaCO_2_ = partial pressure of carbon dioxide; Lac = lactic acid; BE = base excess; PCT = procalcitonin; WBC = white blood cell

Parameter	Patient’s values	Normal range
pH	6.92	7.35–7.45
PaO_2_	85 mmHg	83–108 mmHg
PaCO_2_	63 mmHg	35–48 mmHg
Lac	14.6 mmol/L	0.5–2.2 mmol/L
HCO_3_^-^	10 mmol/L	22–27 mmol/L
BE	−19.7 mmol/L	±3 mmol/L
K^+^	3.1 mmol/L	3.5–5.3 mmol/L
Troponin I	3.54 ng/mL	0–0.4 ng/mL
PCT	0.093 ng/mL	0–0.5 ng/mL
WBC	9.52 × 10^9^/L	4–10 × 10^9^/L
Hemoglobin	105 g/L	130–175 g/L
Platelet	190 × 10^9^/L	100–300 × 10^9^/L
Creatinine	38.2 µmol/L	40–115 µmol/L
Uric acid	85 µmol/L	170–420 µmol/L

**Figure 2:**
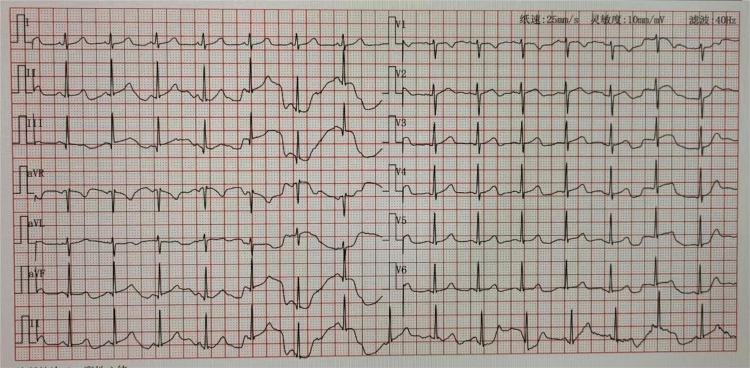
Sinus rhythm on normal electrocardiogram.

On the second day of treatment, the patient’s consciousness transitioned into a shallow coma, with a GCS score of 7T. Her norepinephrine dose was gradually decreased. Routine checking for coagulation suggested a prothrombin time of 26.20 seconds and a prothrombin time of 14.70 seconds, which was thought to be related to the use of heparin in the early stage of treatment. On checking troponin I at 3.54 ng/mL (normal value <0.04 ng/mL) and electrocardiogram (Figure 2), there was no obvious abnormality; The increase in troponin was considered to be related to myocardial injury caused by drugs and low blood pressure in the early stage of treatment.

On the third day of treatment, the patient’s mental state became lucid, and she could breathe on her own, so her ventilator was removed. Her laboratory tests showed no obvious abnormalities. Interestingly, the patient showed a significant increase in urine output, reaching 6,736 mL per day. No obvious abnormalities in renal function were observed. Her urine was noted to have decreased specific gravity, which was considered to be a drug-induced antidiuretic hormone abnormality to be treated with posterior pituitary hormone (0.1 U/kg/minute) to control the volume of urine. She continued to receive blood perfusion to clear the medication, along with the above-mentioned symptomatic treatment.

On the fourth day of treatment, the patient’s urine volume was still high (700-800 mL/hour, 24-hour urine volume = 8,634 mL). Her urine still had a low specific gravity and was clear in color. Therefore, she continued to be treated with posterior pituitary hormone. Her antidiuretic hormone indexes suggested mild reduction, and cranial magnetic resonance imaging to exclude organic lesions did not reveal any obvious abnormality.

**Figure 3:**
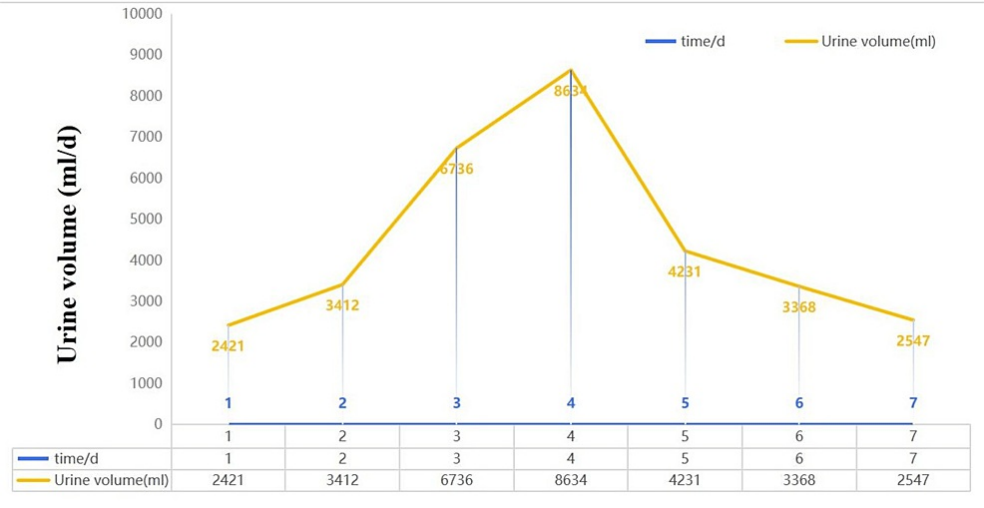
Changes in urine volume during the patient’s hospital stay.

On the fifth day of treatment, the patient’s urine output improved, with a 24-hour urine output of 4,231 mL. Therefore, the posterior pituitary hormone treatment was considered effective, and the dosage was gradually reduced. On the sixth day of treatment, the patient had a 24-hour urine volume of 3,368 mL, indicating further relief and effective treatment. Posterior pituitary hormone treatment was stopped at this time. On the seventh day of treatment, the patient’s 24-hour urine volume was 2,547 mL, representing a normal level. Detailed urine volume data are presented in Figure 3. The patient was then transferred to the general ward to continue treatment.

## Discussion

The secretion and synthesis of vasopressin can be affected by various drugs, such as antidepressants (e.g., SSRIs, tricyclic antidepressants), immune agents (cyclophosphamide), diuretics (hydrochlorothiazide), and oxytocin [9]. Although sertraline hydrochloride is also an SSRI, it rarely appears in clinical treatment, and its related medical history is rarely reported in the literature. Our patient was a 17-year-old young female with a history of bipolar disorder who used to take sertraline hydrochloride tablets, sodium valproate extended-release tablets, and quetiapine fumarate tablets in daily life. She took excessive doses of sertraline hydrochloride this time due to mood swings. Except for sertraline hydrochloride, no other drugs were found in the vicinity when the patient was found, and the level of antidiuretic hormone decreased slightly. These facts support the hypothesis that the diabetes insipidus in this case was associated with excessive sertraline hydrochloride use. Previous studies have found that conventional doses of SSRIs can cause increased levels of antidiuretic hormone, resulting in hyponatremia followed by abnormal antidiuretic hormone syndrome, which can be significantly relieved after a week with drug withdrawal and fluid restriction [10]. The mechanism behind the abnormal occurrence of vasopressin induced by SSRIs remains unclear. Some scholars believe that the drug leads to the decrease of proximal renal tubule sodium reabsorption and a slowed response of the renin-angiotensin-aldosterone system. These factors lead to the continuous increase of urinary sodium excretion, followed by the decrease of water and sodium retention, causing insufficient blood volume, which, in turn, increases plasma levels of vasopressin, leading to abnormal secretion [11].

We found that the patient had obvious polyuria on the third day of treatment, as well as slightly reduced antidiuretic hormone level, which was contrary to our experience of SSRIs causing an increase in antidiuretic hormone level. In the later stage, we will continue to focus on similar phenomena to verify the reduction of vasopressin levels caused by excessive intake of sertraline hydrochloride and to explore the mechanism through animal experiments.

## Conclusions

At present, as the increased prevalence of depression has become a global trend, especially in young patients, the use of antidepressants has gradually become more common. Although it is sometimes reported that SSRIs cause an increase in antidiuretic hormone delivery, our reported case exhibited the opposite effect, namely, a transient decrease in antidiuretic hormone levels. The symptoms of diabetes insipidus last for several days and can be effectively relieved after treatment with pituitrin. Finally, we note that diabetes insipidus may be a rare side effect caused by excessive consumption of sertraline. More data and further studies are needed to clarify the mechanism by which sertraline hydrochloride affects vasopressin, but the therapeutic measures we report have certain clinical significance.
